# Nanotechnology in leukemia: diagnosis, efficient-targeted drug delivery, and clinical trials

**DOI:** 10.1186/s40001-023-01539-z

**Published:** 2023-12-05

**Authors:** Maha M. Salama, Nora M. Aborehab, Nihal M. El Mahdy, Ahmed Zayed, Shahira M. Ezzat

**Affiliations:** 1 Department of Pharmacognosy, Faculty of Pharmacy, Cairo University, Kasr El-Aini Street, Cairo, 11562 Egypt; 2 Department of Pharmacognosy, Faculty of Pharmacy, The British University in Egypt, El Sherouk City, Suez Desert Road, Cairo, 11837 Egypt; 3 Department of Biochemistry, Faculty of Pharmacy, October University for Modern Sciences and Arts (MSA), Giza, 12451 Egypt; 4 Department of Pharmaceutics and Industrial Pharmacy, Faculty of Pharmacy, October University for Modern Sciences and Arts (MSA), Giza, 12451 Egypt; 5 Department of Pharmacognosy, College of Pharmacy, Tanta University, Elguish Street (Medical Campus), Tanta, 31527 Egypt; 6 Department of Pharmacognosy, Faculty of Pharmacy, October University for Modern Sciences and Arts (MSA), Giza, 12451 Egypt

**Keywords:** Leukemia, Nanocarriers, Targeted nanoparticles, Drug delivery

## Abstract

Leukemia is a group of malignant disorders which affect the blood and blood-forming tissues in the bone marrow, lymphatic system, and spleen. Many types of leukemia exist; thus, their diagnosis and treatment are somewhat complicated. The use of conventional strategies for treatment such as chemotherapy and radiotherapy may develop many side effects and toxicity. Hence, modern research is concerned with the development of specific nano-formulations for targeted delivery of anti-leukemic drugs avoiding toxic effects on normal cells. Nanostructures can be applied not only in treatment but also in diagnosis. In this article, types of leukemia, its causes, diagnosis as well as conventional treatment of leukemia shall be reviewed. Then, the use of nanoparticles in diagnosis of leukemia and synthesis of nanocarriers for efficient delivery of anti-leukemia drugs being investigated in in vivo and clinical studies. Therefore, it may contribute to the discovery of novel and emerging nanoparticles for targeted treatment of leukemia with less side effects and toxicities.

## Introduction

Leukemia is a class of cancerous diseases that impact the bone marrow, lymphatic system, spleen, and blood-forming organs. It causes one type of leukocyte, i.e., white blood cell or WBC, to proliferate tremendously resulting in leukocytosis. Leukocytosis is a typical response to infection; but, when it persists over an extended period of time or increases gradually without an apparent reason, it may be a sign of cancer [1]. Leukemia comes in a variety of forms, some of which are more prevalent in young patients, while cases of other types of leukemia affect adults [2]. The most crucial thing is to promptly identify and diagnose affected patients; failing to do so could result in death [3]. Although the precise etiology of leukemia is unknown, a number of variables, including genetic predisposition, chromosomal abnormalities, chemical agents (such as benzene), chemotherapeutic drugs, radiation, immunocompromise, and viruses, may contribute to the disease's development [2].

Over the past few years, malignant cells, including leukemia, have gained resistance against cell division inhibitors and commonly used chemotherapeutic drugs [4]. This resistance suggests that cancer cells may be able to change their genetic makeup to initiate angiogenesis and combat the hypoxia brought on by these agents [5]. Because of this, the latest treatment approaches are made to specifically target the suppression of tumor angiogenesis or alter signaling pathways, which are responsible for the development of chemotherapy resistance and tolerance [6]. Adjuvant and neo-adjuvant surgery, radiation, and combinations of chemotherapeutic drugs are all part of conventional cancer treatment regimens. The side effects and toxicities that come with the use of such protocols include bone-marrow suppression and consequently immunosuppression, inflammation of the mucosa, gastrointestinal (e.g., nausea, vomiting, and diarrhea) in addition to alopecia, exhaustion, sterility, and infertility. In addition, immunosuppression increases the risk of infections [7–9]. Therefore, the focus of current research is on creating customized nano-formulations that deliver drugs to cancer cells specifically while avoiding harmful side effects on healthy cells [10].

Interest in using nanostructures as a vehicle for delivering various medicinal compounds is growing. In addition to being employed as a drug delivery method, nanostructures are also utilized in molecular diagnosis, illness detection, and nanoscale immunotherapy [11, 12]. Target-based drug development regimens, in which the therapeutic agents become specific to the genotype of the cancer cell and possess strong affinity to various molecular targets, were developed by the invention of screening procedures based on nanotechnology [13, 14]. The creation of novel therapeutic techniques, improved drug distribution, and increased bioavailability are the key benefits of using nanoparticles (NPs) in medicine [15]. Numerous NPs types, including dendrimers, graphene, fullerene, metallic, magnetic, polymeric, metal oxide, quantum dots, liposomes, carbon nanotubes, and graphene, may find use in the detection and therapy of cancer [16]. Nanostructures may increase the biologically active drug's transport and deposition at the tumor site, improve therapeutic efficacy, and lessen the severity of side effects on healthy, normal tissues [17]. One can combine a medicinal medicine and a diagnostic agent into a single nanoparticle due to the internal makeup of NPs. In this manner, the drug's release, biodistribution, and accumulation may all be efficiently tracked and measured to evaluate the medication's therapeutic effectiveness [12]. Currently, organic nanomaterials such as liposomes and polymer micelles are the mainstay of Food and Drug Administration (FDA)-approved or clinically researched nanomedicine against hematological malignancies [18].

Prior to the effective clinical use of nano-based anti-leukemia drugs in humans, a number of parameters, including clearance, long-term toxicity, biocompatibility, immunogenicity, selectivity, pharmacokinetics, and biodistribution, still require a lot of investigations. Therefore, the goal of this review is to outline the background that justifies the application of NPs conjugated with various therapeutic agents in targeted therapy and cancer research, with a focus on leukemia specifically. First, we will go over leukaemia: what it is, how it is diagnosed, and what its forms are. Then, the key cellular pathways that are targeted by leukemia medications shall be discussed. The final areas include novel and emerging NPs for targeted leukemia treatment, nanocarriers for efficient anti-leukemia drug delivery, and NPs for leukemia diagnosis. Nano-based easy, rapid, selective, and early diagnosis may provide a clinically valuable information for prompt treatment. The review is devoted for the readers and experts in this important field of medicine, particularly oncology, since the same approaches may be applied for other types of cancers. Create nanomedicines in the future that are resistant to chemotherapy and targeted therapy based on the mechanism which could be the beginning of the end for leukaemia [19].

## Searching strategies

Various scientific databases, including Google Scholar, PubMed, and Scopus, were used, where articles published in peer-reviewed journals and book chapters were considered. The time period was non-specified, but publications were prioritized, particularly in the last 10 years. Keywords related to the main topic of the current research, such as "Leukemia; Nanobiotechnology; Treatment; Diagnosis; Drug delivery". The search resulted in huge number of articles, which have been then filtered to include mainly molecular mechanisms-based studies and advanced formulations for drugs being promoted for in vivo, clinical trials, and approval for diagnosis and management of drug-resistant cases.

### Leukemia types and diagnosis

There are four main forms of leukemia, including acute lymphocytic leukemia (ALL), acute myeloid leukemia (AML), chronic lymphocytic leukemia (CLL), and chronic myeloid leukemia (CML), which are listed in Table 1. Since complicated and distinct group of hematological neoplasms are involved in the diagnosis of leukemia, morphologic variations, cytogenetic anomalies, immunophenotype, and clinical characteristics could distinguish each type from the others [20].

**Table 1:** Major four types of leukemia types and manifestations

Name	Manifestations	References
Acute lymphoblastic leukemia (ALL)	- Malignant transformation and proliferation of the lymphoid cells at an early stage of differentiation which could be able to invade the bone marrow, blood, and extramedullary sites - It represents a devastating disease when it occurs in adults as 80% of acute lymphoblastic leukemia occurs in children	[21, 25]
Acute myeloid leukemia (AML)	- A malignant disorder of haemopoietic stem cells characterized by clonal expansion of abnormally differentiated blasts of myeloid lineage. As a result of this proliferation of immature myeloid cells include accumulation of immature progenitors (blasts) with impairment of normal hemopoiesis, leading to severe infections, anemia, and hemorrhage	[22]
Chronic lymphocytic leukemia (CLL)	- A lymphoproliferative disorder which is characterized by monoclonal, mature CD5^+^ B cells expansion in the peripheral blood, secondary lymphoid tissues and bone marrow - It represents the most common type of leukemia in adults	[26]
Chronic myeloid leukemia (CML)	- A myeloproliferative neoplasm with an incidence of 1–2 cases per 100 000 adults - Predominantly composed of granulocytes, it affects peripheral blood and bone marrow as well - The most common in older adults and men	[3, 27, 28]

The diagnosis of ALL involves cell morphology characterization by microscopy, immunophenotyping which is the gold standard for lineage assessment, classification, and detection of features that are important for the assessment of minimal residual disease. Chromosomal analysis with fluorescence in-situ hybridization or reverse transcription polymerase chain reaction (RT-PCR) is performed for the detection of selected genomic abnormalities, or recently in next generation sequencing have made possible whole genome sequencing [21]. In addition, AML diagnosis requires the identification of 20% or more myeloid blasts with morphological assessment of the peripheral blood or bone marrow. In addition, immunophenotyping by flow cytometry should be investigated to confirm the myeloid origin of malignant blast populations and to categorize the subtype of acute myeloid leukemia. Cytogenetic analysis and screening for the most common occurring gene mutations and rearrangements [22]. However, CLL diagnosis is determined by the presence of more than 5 × 10^9^/l monoclonal B lymphocytes in the peripheral blood. Using flow cytometry, the clonality of these B cells should be verified. Furthermore, the leukemia cells in the blood smear have the typical tiny, mature appearance of lymphocytes, a compact nucleus devoid of distinguishable nucleoli, and partially aggregated chromatin. They also have a limited border of cytoplasm [23]. Besides, the diagnosis of CML can be made using differential excessive agranulocytosis and a distinctive blood count. The detection of the Philadelphia chromosome, *i.e.*, 22q- or BCR-ABL1 transcripts or both, in peripheral blood or bone-marrow cells may be conclusive for CML diagnosis [24].

### Cellular pathways for leukemia drug targeting

#### Canonical nuclear Factor Kappa B (NF-κB)-inducing kinase

**Fig. 1:**
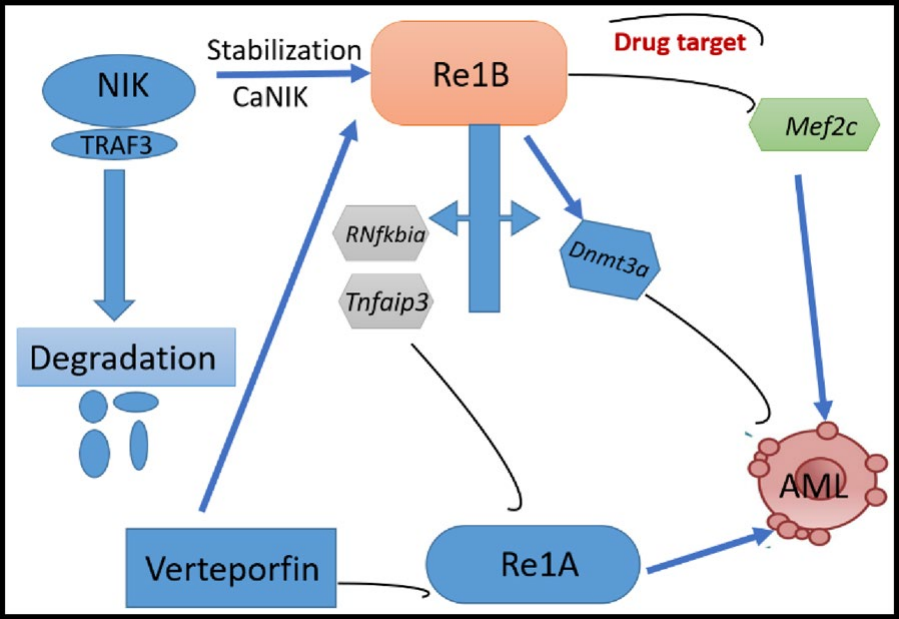
Stabilization of Canonical NF-κB-inducing kinase. AML: acute myeloid leukemia, caNIK: canonical nuclear factor κB inducing kinase; Mef2c: myocyte-specific enhancer factor; Dnmt3a: DNA (cytosine-5)-methyltransferase 3A, RNfkbia: nuclear factor that binds to the enhancer element of the Ig kappa light-chain of activated B cells;Tnfaip3: protein induced by TNF-mediated NF-κB activation and has a dual function in regulating NF-κB associated with inflammatory carcinogenesis in many cancer types; TRAF3: TNF receptor-associated factor

Verteporfin, a benzo porphyrin derivative, evidenced anti-AML by targeting canonical NF-κB—activated in AML stem cells—which is essential for maintenance of the renewal of leukemia stem cells (LSCs). Since, the inhibition of canonical signaling by the ectopic expression of IκBα reduces AML development. Therefore, targeting the pathway is regarded as a potential therapy in AML treatment. Verteporfin impairs NF-κB-inducing kinase (NIK), eventually suppresses AML. It stabilizes NIK, and hence, activates NF-κB non-canonical signaling and inhibits NF-κB canonical signaling. Furthermore, stabilization of NIK-induced activation of NF-κB non-canonical signaling upregulates *Dnmt3a* to deactivate AML and downregulates *Mef2c* that activates AML progression. These upregulations and downregulations of the genes present exceptionally in NIK-stabilized leukemia cell supports the anti-AML of verteporfin [29]. Figure 1 summarizes pathways involved in stabilization of canonical NIK.

#### B-cell receptor (BCR)

The signal that BCR activates is one of the main targets of B-cell tumors. Several potential kinases are released downstream and drive the proliferation and survival of CLL. Consequently, a novel therapeutic strategy for CLL is the development of BCR kinase inhibitors, which specifically target the route of these kinases. A class of drugs known as BCR inhibitors has the ability to stop the target of CLL cells and release them into the peripheral blood stream, where they can be readily attacked by other substances. For instance, ibrutinib and idelalisib are Bruton’s Tyrosine Kinase (BTK) and phosphoinositide 3-kinase-δ (PI3K-δ) inhibitors, respectively, that have been approved for CLL treatment. In addition, duvelisib can inhibit both ẟ and γ isoforms of PI3K. By preventing CLL cells from homing into their specific microenvironments, kinase inhibitors drive CLL cells into the peripheral circulation, where they undergo lymphocytosis without producing hematotoxicity. BCR inhibitors can, therefore, target the CLL cells that are concealed in the lymph node and marrow indentation [30]. Nano-antitumor therapy that targets the tumor microenvironment has gained a lot of attention ascribed to the physiological differences between tumors and normal tissues [31].

**Fig. 2:**
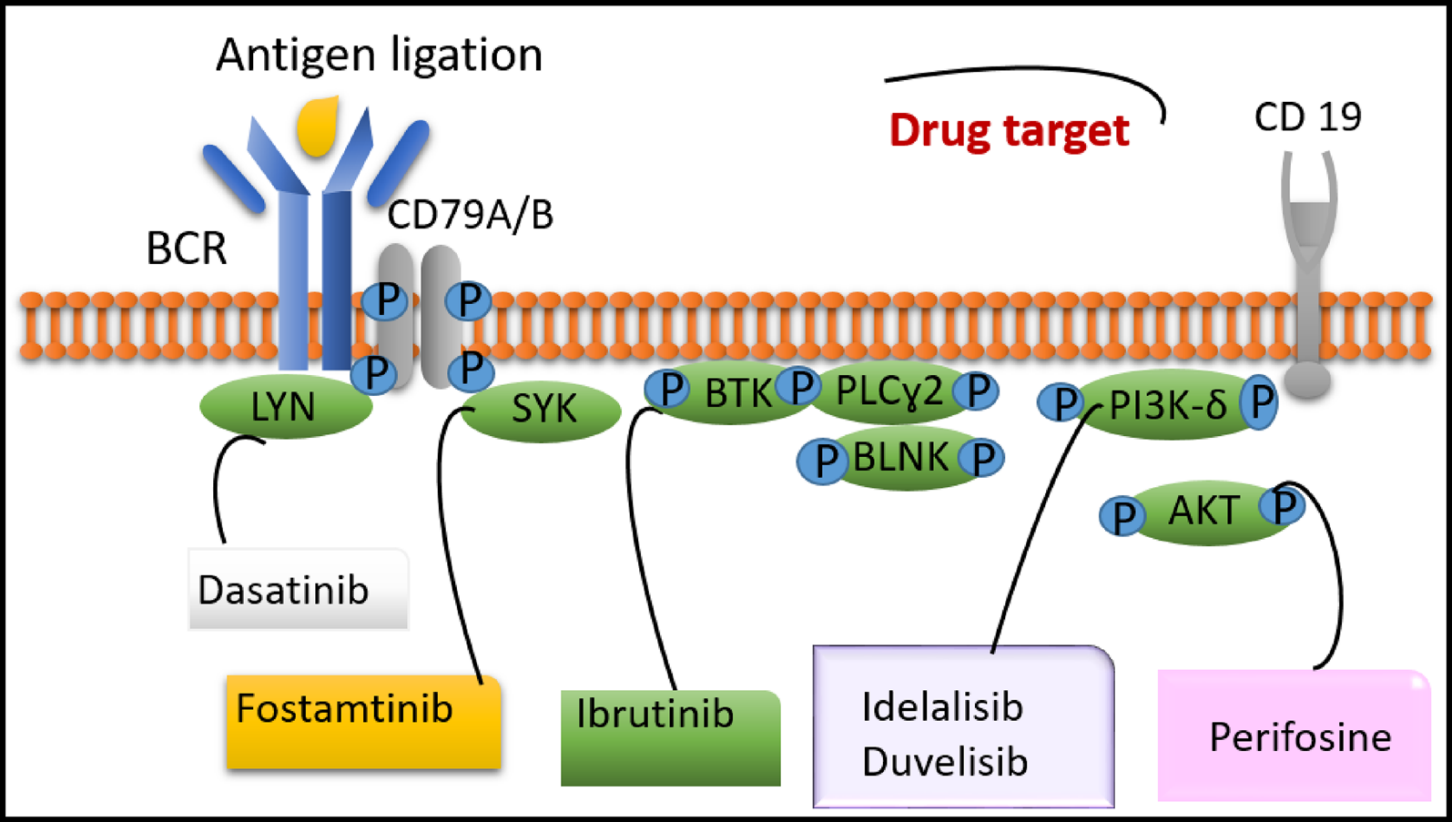
B-cell receptor signaling inhibitors. BCR: B cell receptor; BTK: Bruton’s tyrosine kinase; SYK: spleen tyrosine kinase; BLNK: B-cell linker; PLCγ2: phospholipase Cγ2; PI3K: phosphoinositide 3-kinase, the “P” in blue circle indicates phosphorylation, LYN: a gene on chromosome 8q13 that encodes a non-receptor tyrosine protein kinase, AKT: serine/threonine kinase

Fostamatinib is a spleen tyrosine kinase (Syk) inhibitor, where SYK plays an important role in BCR-mediated survival and is mainly activated in CLL. Therefore, inhibition of SYK signaling avoids the interaction of the CLL cells with its relevant microenvironment and stimulates apoptotic signals. Fostamatinib reduces the phosphorylation of the proximal BCR signaling regulators SLP65 and PLCγ2, in addition to ERK, AKT, and NF-κB downstream pathways, eventually, decreases proliferation and survival of CLL cells [32]. Moreover, perifosine hinders the phosphorylation of Ak strain transforming (AKT) via avoiding the localization of AKT in the membrane, hence interfering with the interaction between PI3K and AKT [33]. The several BCR signaling inhibitors and mediators that are involved are displayed in Fig. 2.

#### Chronic lymphocytic leukemia pathogenesis and the suggested therapeutic targets

**Fig. 3:**
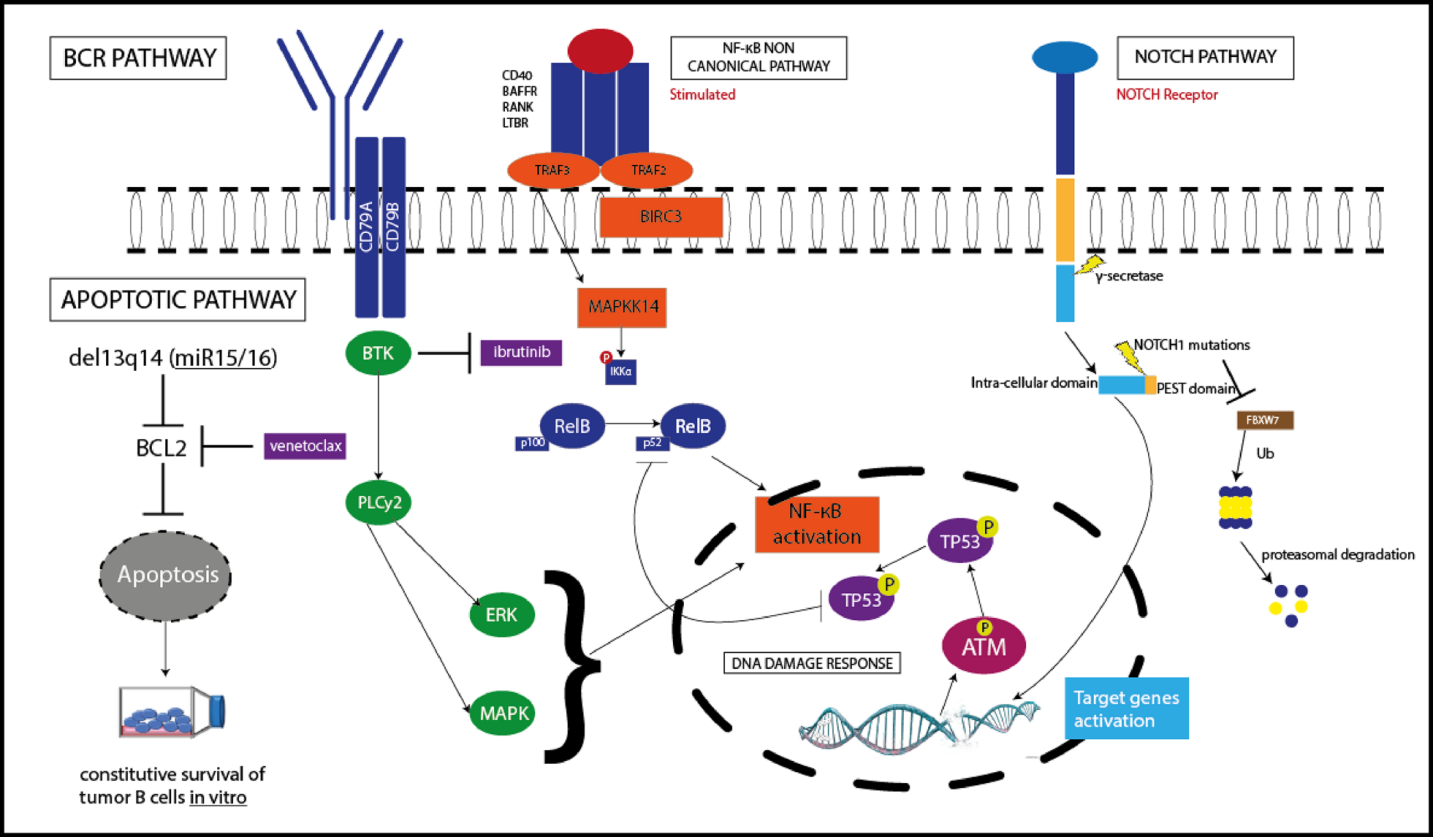
Biological pathways for chronic lymphocytic leukemia pathogenesis and the suggested therapeutic targets. BCL2: B-cell lymphoma 2; BTK: Bruton’s tyrosine kinase; NF-κB: nuclear factor kappa-light-chain-enhancer of activated B cells; (NOTCH1): notch homolog 1 PEST: a peptide *sequence* that is rich in proline (P), glutamic acid (E), serine (S), and threonine (T) of the NICD: NOTCH intracellular domain; TP53: tumor protein 53; ATM: ataxia telangiectasia mutated proteins

Precision medicine plays a backbone in the treatment of CLL, since the integration of molecular biomarkers with the clinical features of the disease may lead to the correct treatment strategy. These treatment strategies depend on certain molecular features of the disease. Tumor protein p53 (TP53) disruption is the strongest prognosticator of resistant cancer, accordingly the assessment of TP53 status’ evaluation is considered the first line treatment. Therefore, patients featuring TP53 disruption need drugs being able to inhibit the BCR or B-cell lymphoma 2 (BCL2) pathway. Moreover, the mutational status of immunoglobulin heavy variable (IGHV) genes directs the modification of treatment. Patients carrying mutated IGHV genes, in the absence of TP53 disruption respond positively with chemo-immunotherapy. Conversely, patients with un-mutated IGHV genes respond negatively to chemo-immunotherapy and require BCR inhibitors [34]. As revealed in Fig. 3, venetoclax can bind to BCL2 and inhibits the anti-apoptotic protein, which is damaged by several molecular mechanisms, such as del13q14, therefore, repairing the apoptosis in CLL cells. Ibrutinib, BTK inhibitor**,** can target BTK which is an essential kinase present downstream to the BCR pathway. Another pathway is the inhibition of the non-canonical NF-κB pathway that is fundamental to cell survival and progression as mutation of upstream signaling molecules TRAF3 leads to activation of non-canonical NF-κB. In addition, NOTCH homolog 1 (*NOTCH1*) mutations dislocate the PEST domain, the NOTCH intracellular domain (NICD), this leads to a vital transcription of target genes enhancing cell proliferation. The canonical activation of Notch signaling is mediated by ligand-mediated mechanisms [35]. Thus, the advance of therapeutic agents that interfere with ligand–receptor binding is regarded as an important pivot in the treatment [36].

#### NOTCH signaling

**Fig. 4:**
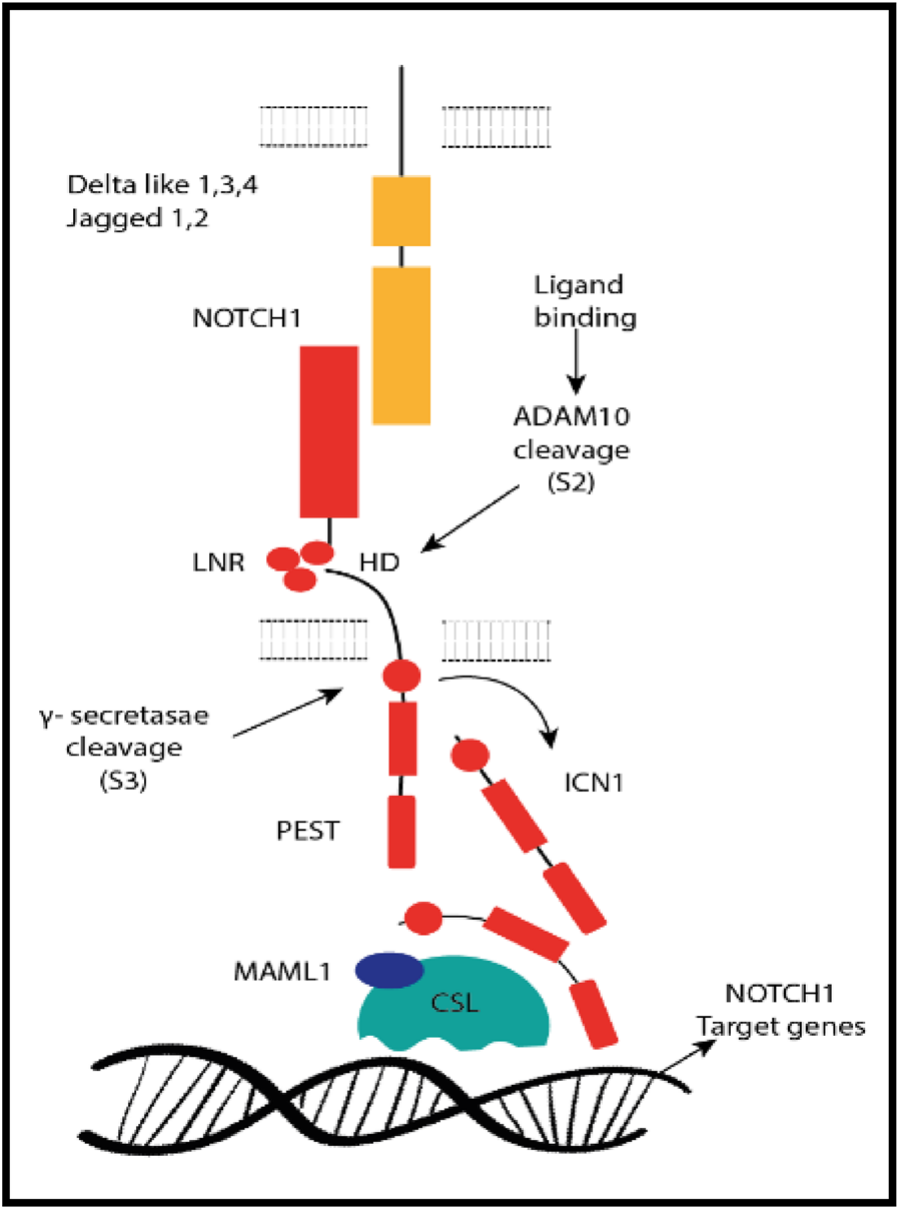
NOTCH signaling pathway. ADAM: metalloprotease (metalloproteinase/disintegrin/cysteine-rich); ICN1: intracellular domains of NOTCH1; MAML1 co-activator (Mastermind-like 1); PEST: peptide *sequence* that is rich in proline (P), glutamic acid (E), serine (S), and threonine (T); HD: heterodimerization domain; LNR: lymph node ratio NOTCH1 activation shows: the interaction between the NOTCH1 receptor, Delta-like and Jagged ligands which are expressed on the surface of a near cells activating the proteolytic cleavage of the receptor; by an ADAM metalloprotease (metalloproteinase/disintegrin/cysteine-rich) (S2 cleavage) followed by the γ-secretase complex (S3 cleavage), which releases the intracellular domains of NOTCH1 (ICN1) from the membrane. ICN1 is transferred to the nucleus and interacts with DNA-binding protein and induces the MAML1 co-activator (Mastermind-like 1) to activate the expression of NOTCH1 target genes

An important active NOTCH1 signaling, initiated by activating mutations in the *NOTCH1* gene, is responsible for some oncogenic transformation that leads to malignant diseases, Fig. 4. T-cell acute lymphoblastic leukemia (T-ALL) is a tumor resulting from the malignant transformation of T-cell progenitors via the mutations in the *NOTCH1* gene. γ-Secretase inhibitors (GSIs) can efficiently block *NOTCH1* signaling in T-ALL, and could be regarded as a targeted therapy [37]. γ-Secretase complex proteolyzes the release of the intracellular portion of NOTCH1 (ICN1) and is considered a crucial step in the canonical NOTCH signaling. Therefore, GSIs that target the NOTCH receptors have been implemented in cancers, where *NOTCH1* mutations are common (e.g., T-ALL and CLL). Studies showed that GSI treatment induces G0/G1 arrest along with rapid clearance of intracellular *NOTCH1*. However, the clinical development of GSIs has been hindered by their low cytotoxicity against human T-ALL and GIT toxicity derived from inhibition of NOTCH signaling in the gut [38]. Furthermore, anti-*NOTCH1* inhibitory antibodies, small peptide inhibitors of NOTCH signaling as well as combination chemotherapy with GSIs and glucocorticoids, have lately been proposed as anti-NOTCH therapeutic protocols. Investigation of NOTCH1 mutations in chronic lymphocytic leukemia has aroused in advance the clinical significance of NOTCH signaling as a therapeutic target in human cancer.

**Fig. 5:**
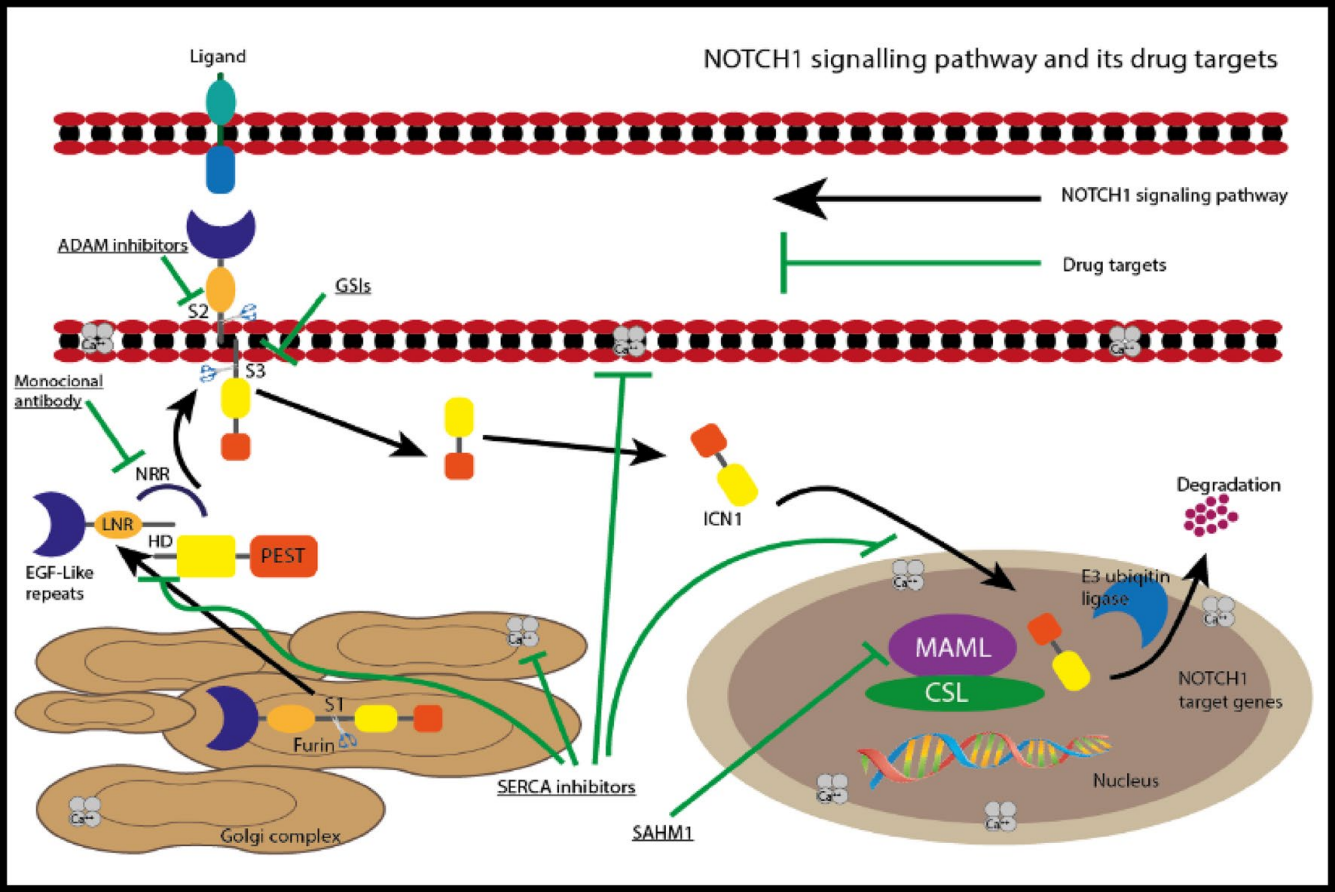
NOTCH1 signaling pathway and drug targets. ADAM: metalloproteinase/disintegrin/cysteine-rich; NRR: negative regulatory region; SERCA: Sarco/Endoplasmic Reticulum Calcium-ATPase; LNR: lymph node ratio; HD: heterodimerization domain; SAHM1: hydrocarbon-stapled synthetic peptide stapled α-helical peptide derived from mastermind-like 1 prevent the formation of MAML: co-activator (Mastermind-like-1); ICN1: intracellular domains of NOTCH1;PEST: peptide sequence is rich in proline (P), glutamic acid (E), serine (S), and threonine (T), CSL: transcription factor. ADAM inhibitors inhibit ADAM protease S2 site uptake; γ-secretase inhibitors (GSIs) prevent the cleavage of S3 site of γ-secretase; Monoclonal antibody prevent the change the spatial arrangement of the negative regulatory region (NRR) from exposing the S2 and S3 cleavage sites; SERCA inhibitors include Ca^2+^ ATPase inhibitors prevent transportation of NOTCH1 and the formation of heterodimerization domain (HD); SAHM1 hinder the binding complex of ICN1–CSL–MAML

One of the hematological malignancies is T-ALL. The novel chemotherapy approaches and/or stem cell transplantation have improved the cure rate for T-ALL; nonetheless, the relapse rate among patients necessitates alternate therapies. Remarkably, one of the main causes of this relapse is gene mutations, specifically NOTCH1 gene mutation. Targeting the NOTCH1 signaling pathway may be crucial to treating relapsed and resistant T-ALL, since NOTCH1 gene mutations are important oncogenes in T-ALL [39]. Hence, A Disintegrin and Metalloprotease (ADAM) inhibitors, gamma secretase inhibitors (GSIs), monoclonal antibody targeting NOTCH1, in addition to sarco/endoplasmic reticulum (ER) Ca^2+^-ATPase (SERCA) inhibitors are potential candidates for leukemia managements. In more details. These classes are illustrated in Fig. 5.

## Conventional treatment of Leukemia

### Chemotherapy

Novel findings of chemotherapeutic agents, which are recognized as scientific and technological advance, allowed insights of cell biology of human cancer cells, and hence, targeted therapy has been applied. Granting the targeted therapy drugs with outstanding accomplishments in certain cancer cells, new therapies still cannot replace cytotoxic agents. Moreover, clinical trials have evidenced the combined effect of targeted molecules and traditional cytotoxic agents [40]. They are classified into alkylating agents (e.g., nitrogen mustards and its based hybrids) which are marketed as cyclophosphamide (Cytoxan^®^ or Neosar^®^) [41].

Purine analogues as 6-mercaptopurine (6-MP) is one of the primary chemotherapeutic agents applied in acute leukemia, and still one of the most convenient drugs in maintenance therapy of ALL [42, 43]. It is marketed under the names Purinethol^®^ and Purixan^®^. In addition, pyrimidine analogues are represented by cytarabine (1-*β*-D-arabinofuranosylcytosine; ara-C) T: Cytosar-U^®^, Tarabine PFS^®^. This drug has been approved by FDA and NCI for the management of ALL, CML and to prevent and treat meningeal leukemia. Its mechanism of action resides in the transportation of ara-C into the cell then it is phosphorylated into ara-C monophosphate (ara-CMP) by DCK and eventually to ara-C triphosphate (ara-CTP) which then contests with deoxycytidine triphosphate (dCTP) for incorporation into DNA and finally blocking DNA synthesis causing cell death [44].

Microtubule-target agents (MTA) have been also involved in the preservation of cell structure, protein transportation and mitosis. *Vinca* alkaloids are potential examples for this class. The most clinically important three alkaloids are vinblastine (VBL), vincristine (VCR), and vinorelbine (VRL) which have been approved in the United states by FDA [45]. Vinca alkaloids are recognized as destabilizing agents, where they depolarize microtubule, block the mitotic progression, and eventually lead to cell death by apoptosis [46]. VCR is marketed as Oncovin^®^ and Vincasar Pfs^®^ and commonly used in combination chemotherapy for treating pediatric leukemia.

Leukemia patients are treated with antibiotics (e.g., anthracyclines and anthracenediones), as one of the main classes of chemotherapeutic drugs. Two anthracyclines for AML, daunorubicin (DD) (Cerubidine^®^) and idarubicin (IDA) (idamycin PFS^®^), are thought to work by forming complexes with DNA through base pair intercalation and inhibiting topoisomerase II activity by stabilizing the DNA–topoisomerase II complex, which stops topoisomerase II from catalyzing the ligation–relegation reaction. Idarubicin may also damage DNA by free radical damage, disrupt the control of gene expression, and limit polymerase activity. Furthermore, it was stated that idarubicin outperformed daunorubicin in terms of intracellular accumulation and DNA binding ability [47, 48]. Besides, mitoxantrone (e.g., Novantrone^®^, Mitozantrone^®^) belong to anthracenediones class and has been approved for use in AML [49]. Notably, compared to anthracyclines, mitoxantrone showed reduced cardiac toxicity because of its lower generation of quinone-type free radicals.

### Combination between DNT cellular therapy with conventional chemotherapy

CD3 + CD4–CD8–double negative T cells (DNTs) are immune cells that produce substantial cytotoxicity in many tumor cells in a nonspecific manner. As a result, they have emerged as functional immune cells in the field of anticancer therapy [50]. The AML resistance to chemotherapy, which ultimately results in recurrence and death, is one of the main problems with conventional chemotherapeutic drugs. To enhance the anti-leukemic effects of conventional chemotherapy and target diseases resistant to chemotherapy, a combination of DNT therapy and chemotherapy was proposed as an alternate treatment. Ara-C and daunorubicin (DNR) were used in combination with DNT in xenograft model, results revealed that the efficacy of combining an adoptive T-cell therapy and low-dose chemotherapy in reducing therapy-resistant AML. These findings suggested utilizing DNTs as an adjuvant cellular therapy following administration of chemotherapy [51].

### Interferon-α (INF-α) in combination with conventional chemotherapy

Imatinib (Gleevec^®^) is a competitive pyridine-based inhibitor interfering with the ATP binding site, including tyrosine kinase, and hence, it is also classified among tyrosine kinase inhibitor (TKI) [52]. This mechanism has qualified imatinib as a drug of choice for the treatment for CML that is evidenced by improvement in patients with CML, though it has limitations, including the inability to eradicate CML primitive progenitors, which resulted in the elevation of the relapse rate when imatinib is withdrawn. On the other hand, interferon-α (IFN-α) (e.g., Intron^®^-A or Roferon^®^-A) directly targets CML stem cells by inducing cytogenetic remission. Consequently, patients treated with IFN-α were able to sustain durable remissions after discontinuing therapy. Nevertheless, the side effects of IFN-α hinder its administration as well as its cost was 200 times those of the conventional therapy [53]. Furthermore, clinical studies suggested that the combination of imatinib and IFN-α is superior to either therapy, due to their different mechanisms of action [54].

### Radiotherapy

Radiation therapy (RD) may be used to eradicate leukemia cells or relieve pain from swollen lymph nodes, the spleen, or the liver. It is also a promising treatment for bone pain brought on by leukemia cells growing in the bone marrow. Low doses of RD may also be administered before stem cell transplantation. It includes external beam radiation therapy (EBRT), total body irradiation (TBI), and total marrow irradiation (TMI) [55]. Because lymphocyte cells are extremely sensitive to radiation, adding RD to systemic therapy has been reported to be effectively used for leukemia treatment. Therefore, use of RD is a well-established regimen prior to allogeneic stem cell transplantation [56].

## Nanotechnology approaches

**Fig. 6:**
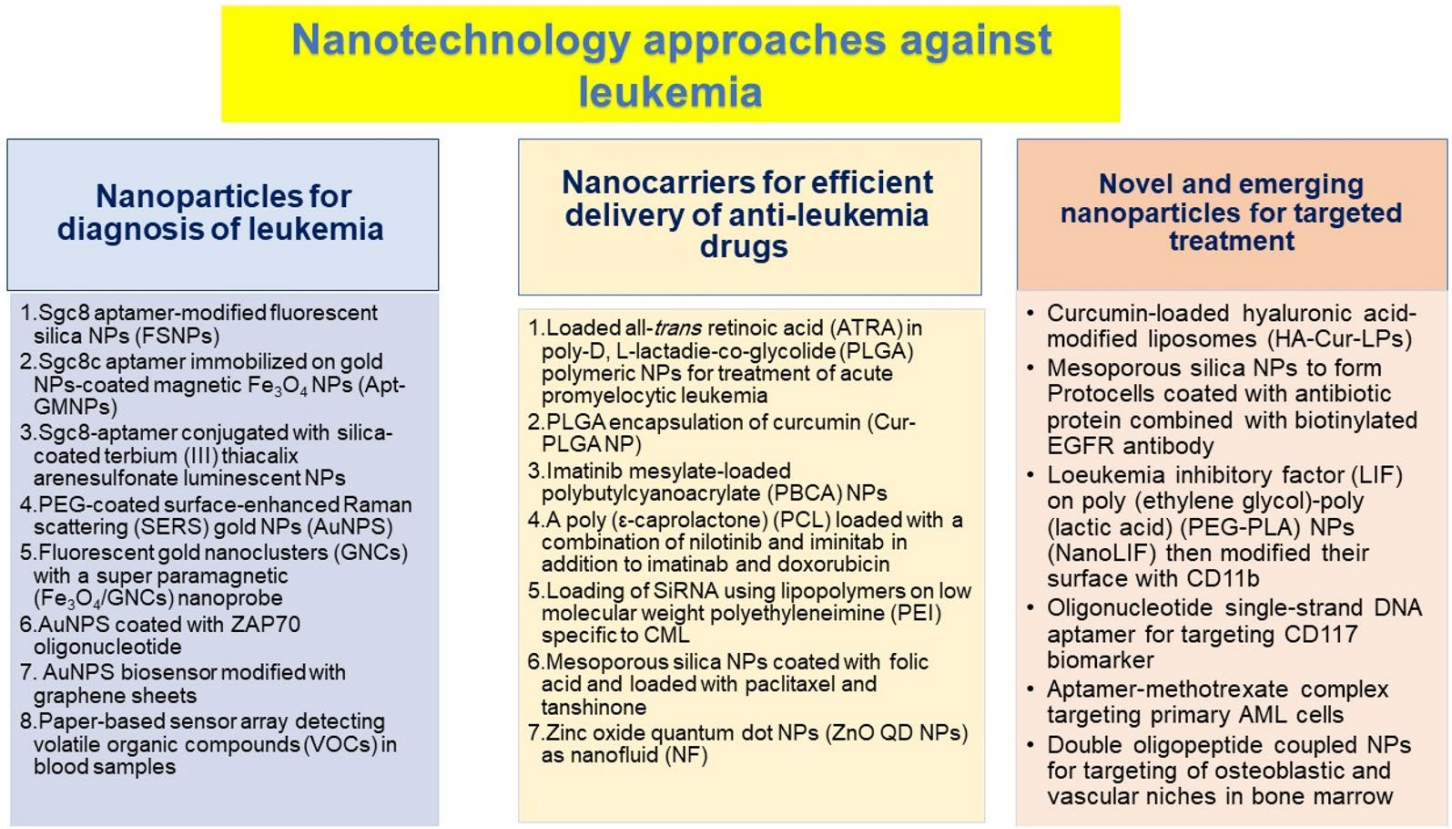
Summary of nanotechnology and nanomedicine approaches applied against leukemia

Nano-oncology and nanomedicine has attracted special interest based on NPs-formulated drugs holding promise for the delivery of small-molecule anticancer drugs especially leukaemia with many trials and studies focusing on the use of NPs for diagnosis and treatment [57, 58]. This interest attributes to the ability of NPs to alter drug solubility and improve their stability in vivo. In addition, they can be tailored to offer specific targeting to specific cells or tissues, thereby reducing the side effects compared to free drugs as well as to overcome resistance to the chemotherapeutics [59–61]. Figure 6 summarizes the various approaches of nanotechnology applied against leukemia. They include diagnosis, drug delivery, and targeted treatment. They shall be also discussed in the following subsections.

NPs with a size range of 1–100 nm are especially beneficent in cancer treatment which offer a large surface area in respect to their volume due to their small particle size which enable the sufficient loading of ligands for targeting of specific cells or organelles delivery as well as stealth such as properties yielded by hydrophilic moieties which protect the encapsulated drug from elimination and extend the drug levels in circulation in vivo thereby retaining their effect for prolonged periods and eliminating frequent dosing [62, 63]. Thus, offering many advantages, such as targeting of drugs, reduced dosage and/or dosing frequency, enhanced drug solubility, increased half-life of drugs in vivo and reduced immunogenicity [64, 65].

### Nanoparticles for diagnosis of leukemia

Traditional detection techniques have certain drawbacks, like being expensive, time-consuming, and labour-intensive. They are not suitable for simple and quick medical monitoring or analysis, since they need a complex combination of equipment, have low sensitivity, and require multiple processing stages. Thus, modern diagnostic tools have been investigated to create cutting-edge tactics in response to the need for the development of improved diagnostic and therapeutic approaches with the highest level of specificity, efficiency, and minimal toxicity [61]. Therefore, rapid, sensitive, easy-to-use, and reasonably priced instruments are becoming more and more necessary for the diagnosis of leukaemia. These tools should not only enable correct diagnosis but also help doctors choose the best course of treatment. Karyotyping, immunophenotyping by flow cytometry or microarray, which combines bone marrow and peripheral blood cytochemical tests, and PCR-based techniques—which are costly and involve numerous steps—are among the diagnostic methods for leukaemia [65]. In this context, NPs have demonstrated their ability to offer substitute approaches to these challenges by offering quick, secure, and precise leukaemia diagnosis techniques.

Specific aptamers which bind to specific target molecules have been widely exploited loading on NPs for detection of leukaemia cells has been applied by many researchers due to their specificity of targeting the desired cells based on loading the NPs with specific targeting molecules with negligible cytotoxicity [63, 66]. One example is Sgc8 aptamer which has been employed in studies because of its selectivity to leukaemia cell. Tan, et al. utilized a simple method to detect leukaemia based on amine-labelled Sgc8 aptamer-modified fluorescent silica NPs (FSNPs) prepared via amide coupling between amino and carboxyl groups which detected leukaemia cells with high accuracy and sensitivity assessed by flow cytometry and fluorescence microscopy. Sgc8-specific targeting combined with FSNPs' showed stronger fluorescence intensity and photostability, and thus, it may provide a clinically valuable tool for early diagnosis and prompt treatment [67]. In addition, Khoshfetrat et al. used thiolated Sgc8c aptamer immobilized on gold NPs-coated magnetic Fe_3_O_4_ NPs (Apt-GMNPs) with a hairpin structure to form Aptasensors and intercalating Ethidium bromide (EB) for the quantification of the leukaemia cells. When leukaemia cancer cells are introduced onto the Aptasensors, the hairpin structure of the aptamer is disrupted and the intercalated molecules are released, resulting in a decrease of the electrochemical signal which is detected. This new diagnostic tool showed a linear response and was successful in detection of leukaemia cancer cells in complex media, such as plasma, without significant interference [68].

Grechkin et al. also utilized Sgc8-aptamer conjugated with amino- and carboxyl-modified silica-coated terbium (III) thiacalix arenesulfonate to form luminescent NPs that were able to detect leukaemia cells using fluorescence microscopy without induction of cell apoptosis or necrosis, which can be common in bioimaging applications. At this experiment, *CCRF*–CEM (human T lymphoblasts cell line) and Jurkat cells (an immortalized line of human T lymphocyte) were investigated as models. The findings indicated that *CCRF*–CEM had a larger shift in fluorescence intensity than Jurkat cells, which may be related to the latter's lower concentration of a tyrosine protein kinase 7 (PTK7) on the cellular membrane [69].

The labelling of NPs or receptor ligands by fluorescent markers is a well-established method for cancer diagnosis as well as the identification of cell phenotype. Maclaughlin et al. harnessed this strategy by developing an antibody-targeted, PEG-coated surface-enhanced Raman scattering (SERS) gold NPs (AuNPS) which can be used to label three cell surface markers on cancerous B cells from the LY10 lymphoma cell line. Using confocal Raman mapping and SERS from cell suspensions, the specificity of the particles' cell labelling was shown on primary CLL and LY10 cells, respectively. The results were also confirmed through the flow cytometer which was used to validate the binding of SERS probes to LY10 over vast cell populations [70]. Moreover, Haghighi et al. combined fluorescent gold nanoclusters (GNCs) with a super paramagnetic (Fe_3_O_4_/GNCs) nanoprobe for imaging and detection of human leukaemia cancer cells (HL-60) using (γ-Mercaptopropyl) trimethoxysilane (MPS) as a stabilizer decorated with the thiol-modified KH1C12 aptamer. The aptamer-functionalized Fe_3_O_4_/GNCs nanoprobe proved its ability to uptake and -image HL-60 cancer cells as well as detection of cancer cells from a range of concentrations 10–200 cells μL^−1^ which can help in early diagnosis of lekemia. The nanoprobe's magnetic and fluorescent properties are particularly significant for MRI-based fluorescence imaging and the collection of HL-60 cancer cells, which may aid in the early detection of extremely aggressive human leukaemia [71]. Asides, AuNPS were fabricated as biosensors by Ensafi et al. by modification of a gold electrode with AuNPS coated with ZAP70 oligonucleotide probe to detect specific sequence of ZAP70 gene, which is a predictor of the IgVH mutation status and serve as prognostic indicator for distinguishing Ig-mutated from Ig-unmutated CLL. The biosensor can detect with a detection limit ZAP70 DNA sequence of 4.0 × 10^−15^molL^−1^ and a decent calibration range between 2.0 × 10^–14^ and 1.0 × 10^−9^molL^−1^. This biosensor is thought to be the most informative stage-independent prognostic indicator for CLL patients [72]. Another application of AuNPS was done by Mazloum-Ardakani et al. to synthesize a stable composite with electrical properties, on the surface of a biosensor by synthesizing poly (catechol) with enhanced conducting properties by modification with graphene sheets and loading on AuNPS, i.e., made up of conductive polymers and nanoparticles. With a detection limit of 1.0 pM for the DNA strand, the peak currents of the electroactive probe catechol were linearly correlated with the logarithm of the target DNA concentrations in the range of 100.0 μM to 10.0 pM. This study looked into a novel, stable composite that was used to identify AML [73].

Furthermore, Bordbar et al. utilized a different and simple technique by employing volatile organic compounds (VOCs) in blood samples as biomarkers to diagnose leukemia based on a paper-based sensor array for detecting leukemia which contains 16 NPs deposited on a sheet of hydrophobic paper in a 4 × 4 array format. The diagnosis was made by comparing the image of sensors recorded by a scanner before and after exposing to the blood vapour. The accuracy of the rock curve in differentiating patients from the control group was 96%. According to the logistic regression model, this approach properly categorised 93.6% of healthy individuals and 93.2% of patients. In addition, for every 20 units rise in overall response, the likelihood of being classified as a patient fell by 10%. With improved sensitivity and specificity, it is a low-cost, non-invasive method of identifying new leukaemia cases [74]. Recently, Wang et al. have reviewed the use of iron oxides NPs which have gained increasing attention in diagnosis and treatment of leukemia. The ability to detect magnetic resonance imaging (MRI) as effective contrast agents, magnetic biosensors, and targeted administration of anti-leukemia medications by coating various targeting moieties are among the benefits of iron oxide nanoparticles [75].

### Nanocarriers for efficient delivery of anti-leukemia drugs

The application of NPs and nanocarriers has improved the delivery of chemotherapeutic agents, primarily to lessen their toxicity to normal tissue. Other benefits include improving the bioavailability, reducing side effects, solubility, blood circulation time. In addition, anti-tumor drug candidates were found to be improved by nano-delivery systems, which also use targeting or sensing techniques to release medications selectively at specific locations [61, 64, 76]. The same is true for anti-leukemia drugs, which is why many research have looked into different ways to distribute them using nanocarriers.

Polymeric NPs are one example of nanocarriers widely used by researchers for loading of drugs due to their versatility and potential low toxicity [77]. Simon et al. loaded all-*trans* retinoic acid (ATRA) in poly-D, L-lactadie-co-glycolide (PLGA) polymeric NPs for treatment of acute promyelocytic leukemia. This strategy allowed ATRA to be administered intravenously instead of orally which is valuable for patients who have difficulties in swallowing, which is common in case for leukemia patients [78]. PLGA was also utilized by Leung et al. for encapsulation of curcumin (Cur-PLGA NP) using a microfluidic-assisted nano-precipitation surfactant free technique that showed excellent colloidal and drug stability as well as a dose-dependent cytotoxicity in leukemia Jurkat cells without altering the viability of fibroblast NIH3T3 cells [79]. Hasandoost et al. prepared imatinib mesylate-loaded polybutylcyanoacrylate (PBCA) NPs which enhanced the cytotoxic effects of imatinib mesylate compared to free drug alone. The miniemulsion polymerization process was used to create the formulation demonstrating that the drugs were physically entrapped by nanoscale particles with a high encapsulation efficiency of 86% [80].

Combined therapy is often used in leukemia treatment to avoid resistance to treatment. Cortese et al. research team focused on this strategy and benefited from NPs ability to load several drug molecules by formulating a poly (ε-caprolactone) (PCL) system with biodegradable pH-sensitive core for nilotinib release and an enzymatic sensitive outer shell for imatinib release which allowed for combined therapy of both drugs. This system showed better cytotoxicity to leukemia cells compared to free drug [81]. The same team used the PCL loaded NPs with imatinib in combination with doxorubicin loaded sensitive polyelectrolyte complexes (PECs) which allowed a down-regulation of BCR–ABL in a sustained manner as well as significant CML stem cell death as both drugs worked in a synergistic manner with maximized cytotoxicity with minimized probability of cell resistance to any of these drugs [82]. The same research team also utilized PCL NPs to encapsulate RNA–protamine complex encapsulated for the intracellular delivery of mRNA molecules. These NPs had a core–shell structure with an mRNA-containing inner core surrounded by PCL layers which increased the in vivo stability of the mRNA and allowing their efficient delivery [83].

The loading of SiRNA was attempted by Valencia-Serna et al. using lipopolymers, namely, α-linolenic acid (ALA) substitution on low molecular weight polyethyleneimine (PEI) to deliver specific siRNAs to CML cells for therapeutic gene silencing of BCR–ABL gene. The results showed that siRNA/PEI–ALA NPs successfully allowed the silencing of the BCR–ABL gene and BCR–ABL protein, thereby decreasing the growth on CML K562 cells in vitro and arresting the growth of localized tumors in a CML mouse model [84].

Inorganic NPs have been widely exploited as nanocarriers for leading of ant-leukemic drugs. Mesoporous silica NPs were coated with folic acid PEG–lipid bilayer membrane loaded with paclitaxel and tanshinone by Li et al. It allowed a sustained release of both drugs with enhanced uptake of the NPs by NB4 cells due to the presence of folic acid leading to targeting of folic acid receptors on NB4 cells [85]. Drug resistance was diminished by Deng et al. through combined delivery of AS1411, doxorubicin and anti-221 via multifunctional AuNPS which significantly decreased the proliferation and clonogenic potential as well as facilitating the apoptosis of drug resistant leukemia cells. These systems also showed marked down regulation of miR-221 and DNMT1, leading to restoring the expression of p27kip1 and p15ink4b tumor suppressors as well as miR-221-mediated decrease of P-glycoprotein (P-gp) expression thereby providing a more efficient treatment [86]. AuNPS were also utilized by Petrushev et al. to conjugate TKIs that inhibit FLT3 kinase thereby targeting the FLT#3 mutation causing relapse in AML patients. The new prepared bioconjugate had better therapeutic effect that TKI alone as well as effective trans-membrane delivery [87].

Carbon-based NPs have been utilized due to their biocompatibility and high loading efficiencies. Shahiri.et al. synthesized multi-walled carbon nanotubes (MWCNTs) coated with silver NPs (Ag-NPs) which showed efficacy as antioxidant and cytotoxic agents against AML and acute T-cell leukemia cell lines in a dose depended manner [88]. Moreover, Felix et al. formulated imatinib loaded quantum dots, which graphene NPs are known for their safety and internalization efficiency in the cells. The results of this research confirmed the ability of the quantum dots loaded with Imatinib to sufficiently internalize and induce apoptosis and killing of cancer cells [89]. Quantum dots were also used by Esmaeili et al. as zinc oxide quantum dot NPs (ZnO QD NPs) as nanofluid (NF) to treat acute promyelocytic leukemia (APL)-derived NB4 cells. The resultant NPs successfully lowered the proliferation of NB4 cells through p21-mediated G1 cell cycle arrest and enabled their apoptosis via reactive oxygen species (ROS)-dependent up regulation of FoxO3a and SIRT1 as well as peroxisome proliferator-activated receptors gamma (PPARγ) up regulation in NB4 cells leading to elevated expression of pro-apoptotic molecules in NB4 cells [90].

Furthermore, Anu et al. synthesized selenium nanoparticles (SeNPs) as green biogenesis NPs from *Cassia auriculata* and shown their effectiveness against leukemia cancer cells in vitro, as well as greater solubility and decreased toxicity on normal cells [91]. Niosomal formulations to encapsulate ifosfamide, alkylating anticancer drug, was developed to control its release and alleviate its toxicity [92]. Nanocarriers for efficient delivery of anti-leukemia drugs are summarized in Table 2.

**Table 2:** Nanocarriers for efficient delivery of anti-leukemia drugs

Drug	Nanocarrier	Results	References
ATRA	PLGA polymeric nanoparticles	Allowed ATRA to be administered intravenously instead of orally	[78]
Curcumin		Excellent colloidal and drug stability as well as a dose-dependent cytotoxicity in leukemia Jurkat cells without altering the viability of fibroblast NIH3T3 cells	[79]
Imatinib mesylate	Polybutylcyanoacrylate (PBCA) nanoparticles	Enhanced the cytotoxic effects of imatinib mesylate	[80]
Nilotinib Imatinib	PCL nanoparticles	Better cytotoxicity against leukemia cells compared to free drug	[81]
Imatinib and doxorubicin		Sustained down-regulation of BCR–ABL as well as CML stem cell death due to synergism of both drugs with maximized cytotoxicity and minimized resistance to any one drug	[82]
RNA–protamine complex	PCL nanoparticles	High stability and stealth properties	[83]
SiRNA	(α-LA)–PEI nanoparticles	Silencing of the BCR–ABL gene and BCR–ABL protein, thereby decreasing the growth on CML K562 cells and arrested the growth of localized tumors	[84]
Paclitaxel and tanshinone	Mesoporous silica nanoparticle	A sustained release of both drugs with increased the uptake by NB4 cells compared to free drug	[85]
AS1411, doxorubicin and anti-221 miRNA	Gold nanoparticles (AuNPS)	Restored tumor suppressor expression and reduction of P-gp expression	[86]
Tyrosine kinase inhibitor		The new prepared bioconjugate had better therapeutic effect that TKI alone as well as effective trans-membrane delivery	[87]
-	Silver nanoparticles loaded on multi-walled carbon nanotubes	Increased antioxidant activity against leukemia cells in dose dependent manner	[88]
Imatinib	Quantum Dots	sufficient internalization and apoptosis of cancer cells	[89]
ZNO		Lowered proliferation of NB4 cells through p21-mediated G1 cell cycle arrest and induced apoptosis leading to elevated expression of pro-apoptotic molecules	[90]
*Ziziphora clinopodioides* Lam leaf aqueous extract	Cadmium nanoparticles (CdNPs)	Ameliorated significantly (*p* ≤ 0.01) the biochemical, inflammatory, RBC, WBC, platelet, stereological, histopathological, and cellular–molecular parameters (anti-acute myeloid leukemia)	[93]

### Novel and emerging nanoparticles for targeted treatment

Several efforts have focused on the development of approaches that allowed targeted delivery of chemotherapeutic drugs as most of these drugs lack selectivity which cause major side-effects as well as lower effectiveness in many patients. Thus, the need arises to smart drug formulations that deliver the drugs selectively to cancer cells, thereby enhancing drug efficacy and reducing nonspecific cytotoxic effects to normal cells [59, 94]. The novel and emerging NPs for targeted treatment of leukemia are summarized in Table 3.

**Table 3:** Novel and emerging nanoparticles for targeted treatment of leukemia

Type of nanoparticle	Targeting mechanism	References
Curcumin loaded hyaluronic acid–liposomes (HA-Cur-LPs)	Targeting of the CD44 receptor thereby targeting curcumin to these cells with high stability	[95]
Hyaluronic acid-coated silver nanoparticles (HA-AgNPs)	Targeting CD44 receptor on leukemia cells	[96]
Gemcitabine loaded Mesoporous silicon nanoparticles coated with lipid bilayer	Coating with biotinylated EGFR antibody achieved leukemia cells targeting as well as offering a targeted release of gemcitabine	[97]
Leukemia inhibitory factor (LIF) on (PEG–PLA) nanoparticles (NanoLIF)	Modified their surface with CD11b antibody to target activated peripheral macrophage	[98]
Arsenic trioxide loaded double oligopeptide coupled nanoparticles	Enhanced the stability and loading efficiency of Arsenic trioxide as well as targeting in the lesions	[100]
Aptamer–methotrexate conjugate	Targeting the biomarker CD117, which is over expressed on AML cells with no effect for the background marrow cells	[99]
siRNA loaded folate–PEG oligoaminoamide,	Targeted delivery as folic acid receptor is over expressed in leukemia cells	[101]
CRISPR/Cas9 system loaded PLGA–PEG nanoparticles	Encapsulating a CRISPR/Cas9 plasmid (pCas9) expressing gRNA targeting the BCR–ABL gene (pCas9/gBCR–ABL) in CML cells without affecting normal cells	[102]
Gold nanoparticles with an antisense oligonucleotide	Silencing the BCR–ABL1 chimeric gene enhanced leukemia cell apoptosis	[103]
Exosomes loaded with miR-365 siRNA	Successfully endocytosed and accumulated SiRNA into CML cells	[104]
Cyclodextrin nanoparticles decorated with FAB antibody to targeting AML	Targeting LSC surface antigen IL-3 receptor α-chain	[105]
Transferrin paclitaxel loaded lipid nanoparticles (TPLN)	Transferrin targeting HL-60 cancer cells	[106]
Amino-PEGylated quantum dots (QDs) and coated with octreotide	Targeting for SSTR receptor which is highly expressed on the surface of leukemia cells	[107]
A fluorescent lanthanide oxyfluoride nanoparticle-based multifunctional peptide drug delivery system and conjugated for treatment AML	Targeting MDM2 and/or MDMX by a dodecameric peptide antagonist (PMI) and an overexpressed cell surface receptor, CD33 through humanized monoclonal antibody	[108]

One strategy of targeting is to prepare or coat the NPs with a specific target for an over-expressed receptor on a cancer cell. Sun et al. developed curcumin loaded hyaluronic acid-modified liposomes (HA-Cur-LPs) which targeted the CD44 receptor and consequently curcumin is targeted to these cells with high stability as CD44 is overexpressed on the surface of leukemia cells. HA-Cur-LPs also provided mice with longer survival time compared to free curcumin treatment [95]. Hyaluronic acid was also utilized by Zhang et al. through formulating hyaluronic acid-coated AgNPs (HA-AgNPs) via electrostatic interaction between the negatively charged HA molecules and positively charged AgNPs which exert their anti-leukemic action through ROS overproduction thereby inhibited the leukemia cells viability by specific binding of HA with CD44 receptors causing apoptosis while having decreased systemic toxicity [96].

Mesoporous silica NPs were used by Durfee et al. used to form a template surrounded by a liposome layer on them to form Protocells. These Protocells were then coated with antibiotic protein combined with biotinylated EGFR antibody. The prepared NPs-targeted leukemia cells efficiently as well as offering a targeted release of gemcitabine which prevented proliferation of myeloid leukemia stem cells and induced apoptosis [97].

Davis et al. loaded leukemia inhibitory factor (LIF) on poly (ethylene glycol)–poly (lactic acid) (PEG–PLA) NPs (NanoLIF) then modified their surface with CD11b antibody to target activated peripheral macrophage. The resultant NPs significantly decreased M1 cell proliferation over 72 h compared to free LIF [98].

Another strategy is oligopeptides and oligonucleotides which can be synthesized to target specific biomarkers or even synthesize personalized drugs for individual patients. Zhao et al. formulated an oligonucleotide single-strand DNA aptamer for targeting CD117 biomarker, which is over expressed on AML cells. Furthermore, they also designed an aptamer − methotrexate complex which could target primary AML cells without affecting the background marrow cells [99]. Fan et al. synthesized a double oligopeptide coupled NPs for targeting of osteoblastic and vascular niches in bone marrow. The prepared NPs also enhanced the stability and loading efficiency of Arsenic trioxide and targeted it to the cancer lesions eliminating primitive CML cells as well as improving arsenic trioxide protection to the original CML cells, and reducing the size of the K562–HUVEC organoid [100].

RNA-based therapies were exploited for treatment of leukemia as they can silence gene expression, express proteins, edit genes, thereby serving as a tailored treatment for specific types of leukemia; however, they suffer from poor in vivo stability and thus need to be encapsulated in NPs which have the stealth ability thereby preserving them from degradation [59]. Lee et al. loaded siRNA polyplexes on folate–PEG oligoaminoamide that protected siRNA against degradation as well as targeted its delivery to leukemia cells as it has folic acid receptor over expression. [101]. Lui et al. targeted a CRISPR/Cas9 system for treatment of CML by encapsulating a CRISPR/Cas9 plasmid (pCas9) which expresses gRNA to target the BCR–ABL gene (pCas9/gBCR–ABL) with poly(ethylene glycol)-b-poly(lactic acid-co-glycolic acid) (PEG–PLGA)-based cationic lipid-assisted polymeric NPs (CLANs). The prepared NPs were able to disrupt the CML-related BCR–ABL gene without affecting the same gene in normal cells thereby improving the survival rate in a CML mouse model [102]. AuNPS decorated with an antisense oligonucleotide were used for silencing the BCR–ABL1 chimeric gene and enhancing leukemia cell apoptosis significantly with loss of viability of imatinib-resistant K562 cells [103]. Exosomes are natural vesicles secreted by cells were utilized by Min et al. through extracting exosomes from imatinib-resistant CML cells and using them as vectors to deliver miR-365. Successful uptake of exosomes CML cells was achieved as well as successful targeted receptors on target cells, thereby accumulating miRNA in target tissues [104].

Cyclodextrin NPs were synthesized by Guo et al. and decorated with FAB antibody to target AML which specifically targets human leukemia stem cells (LSC) surface antigen IL-3 receptor α-chain. Furthermore, it had a synergistic therapeutic effect when combined the drug ara-C [105]. Dai et al. developed transferrin paclitaxel loaded lipid NPs (TPLN) which showed enhanced targeting to the HL-60 cancer cells compared to that of the paclitaxel-loaded NPs (PLN) due to the ability of transferrin to target leukemia cells leading to remarkable apoptosis of leukemia cells [106].

Abdellatif et al. utilized quantum dots, a form of carbon-based NPs to formulate amino-PEGylated quantum dots (QDs) which were succinylated using succinic anhydride and coated with octreotide (QD–OCD) to target somatostatin receptor type-II (SSTR) which is expressed in leukemia cells. QD–OCD accumulated in blood cells indicated by their fluorescence intensity due to SSTR targeting thereby showing potential for leukemia treatment [107]. In addition, Nui et al. synthesized a fluorescent lanthanide oxyfluoride nanoparticle-based multifunctional peptide drug delivery system for AML treatment via targeting mouse double minute 2 (MDM2) and/or MDMX by a dodecameric peptide antagonist (PMI) and an overexpressed cell surface receptor, CD33 through humanized monoclonal antibody. The resultant nanoparticle had no toxicity on normal cells and enabled apoptosis induction of AML cell lines and primary leukemic cells. An additional advantage of the prepared NPs is their fluorescence which facilitated real-time visualization of apoptotic events in AML cells, which is useful in tracking the response and treatment efficiency [108].

Finally, inflammation and bacterial oral infection may accompany and worsen the case of leukemia patients as a result of chemotherapy and immunosuppression. A bimetallic clusterzyme was created by Pt atom replacement in the structure of gold nanocluster (Au/Pt NCs) coupled with glucose oxidase (GOX) Au/Pt NCs@GOX). The in vivo investigations demonstrated that it is possible to successfully cure periodontitis in rats, and enhance periodontal tissue regeneration [109]. In addition, a modified photocross-linkable gelatin-based polymer (GelMA) containing cationic quaternary ammonium salt groups may be helpful in preventing accompanied infections [110]. The associated coagulation and hemostasis problems should be investigated by synthesis of biocompatible anticoagulant and hemostatic materials through surface-functionalized designs [111].

### Clinical trials

Using carefully designed materials to create innovative treatments and devices that may lessen toxicity while improving treatment delivery and efficacy is known as nanotechnology in oncology. Notably, the most well-known nano-based medications, Abraxane^®^ and Doxil^®^, were approved by the FDA a few years ago and have since been effectively employed in clinical settings. Currently, various nano delivery systems have undergone experimental stage and animal models. However, many factors need to be established before its successful clinical applications on humans, such as clearance, long-term toxicity, biocompatibility, selectivity, immunogenicity, pharmacokinetics and bio distribution which still lake sufficient data [112]. Nevertheless, many NPs drug products have gone successfully through clinical trials or even gained approval for marketing.

One example is Vyxeos^®^ by Jazz pharmaceuticals which obtained FDA approval in 2017 which is a liposome injection loaded with daunorubicin and ara-C. This drug product utilized the advantage of loading several drugs in the same carrier or vesicle, offering the advantage of combined therapy which is beneficial for treatment of leukemia compared to a single drug therapy and consequently offered a significantly increased survival rate [113]. Marqibo^®^ is another FDA approved drug product comprising sphingomyelin and cholesterol liposome NPs loaded with VCR. The formulations aim to eliminate the limitations of dosing and pharmacokinetics of conventional VCR drug products in addition to increase the circulation time, optimized delivery to target tissues and enhanced dosing concentrations without increasing toxicity. In addition, patients treated weekly with this new formula achieved complete remission of 20% with an overall response rate of 35%. This new formulation also counteracted the dose-dependent toxicity of VCR administration, such as neuropathy [114].

According to clinicaltrials.gov, current clinical trials involve a study done in University Hospital Zurich, Switzerland for the use of magnetic NPs, coated with epithelial cell adhesion molecule (EpCAM)–antibodies to target epithelial tumor cells. The study aims to see if circulating tumor cells (EpCAM or CD52 as marker) can be removed out of the blood with the help of these particles for the aim of leukemia diagnosis. Another study is conducted by AstraZeneca in phase I/II to determine the maximum tolerated dose as well as the safety, pharmacokinetics, and pharmacodynamics of AZD2811 NPs either as single therapy or in combination with other drugs for treatment of relapsed/refractory AML patients or for treatment of naïve AML patients who are not eligible for intensive induction therapy.

Currently in phase I, Memorial Sloan Kettering Cancer Centre in collaboration with National Cancer Institute (NCI) and Actinium Pharmaceuticals are conducting a clinical study on Targeted Atomic Nano-Generators (Actinium-225-Labeled Humanized Anti-CD33 Monoclonal Antibody HuM195) to determine the safety and dosing of actinium-225 when it is labelled to HuM195. The medication is acknowledged as a clinical-stage radioimmunotherapy that targets CD33 and has demonstrated single-agent activity in AML treatment. The result of this research revealed that its combination with venetoclax is a potential candidate for the treatment of patients with venetoclax-resistant AML [115].

A phase I study by Mayo clinic and national cancer institute currently investigates the safe therapeutic doses as well as the adverse effects of paclitaxel albumin–nanoparticle formulation (nab-paclitaxel) and rituximab-coated nanoparticle for treatment of unresponsive or relapsing B-cell non-Hodgkin lymphoma (NHL) in patients.

Despite of the significant advancements in the field of nanomedicine, the potential harmful effects of nanomaterials on humans remain a source for concern. Significant studies have been performed in recent years to try to understand these harmful effects, and as a result of cellular exposure to nanomaterials, a number of pathways, mechanisms, and important influencing parameters have been linked. The immunological changes brought on by these nanoparticles have also been clarified by these assessments of nanotoxicology. Understanding the process by which nanomaterials trigger immunological responses is necessary to regulate the immunomodulatory effect of these materials in applications [116].

## Conclusion, limitations, and future perspectives

Frequent dosing, serious side effects, and a lack of specificity in anticancer medications are all part of the traditional chemotherapy for leukemia. Consequently, devising a plan to administer the anti-leukemic medication straight to the cancerous location. NPs have recently shown promise in the diagnosis and treatment of many forms of leukemia due to their easy manufacturing process and ability to encapsulate medications with a variety of chemical compositions. In addition to their capacity for sustained drug release and intracellular delivery, NPs for the treatment of leukemia are distinguished by their capacity to reduce the toxicity of the drug on healthy, normal tissues. Hence, to demonstrate the safety and selectivity of such nanotechnology, additional clinical trials will be necessary before it can be used to treat leukemia.

Through the use of smart NPs, the field of nanomedicine has steadily advanced over the past few decades in the accurate diagnosis and focused treatment of many cancers. However, before these advancements can completely replace traditional cancer treatment methods, such as chemotherapy and/or radiation therapy, they must first show in clinical trials that they are both safe and effective. Despite that most of these nano-formulations have shifted to target and deal with solid tumors, aiming to influence their ability to metastasize, spread, implantation, and trigger an inflammatory response. Later, novel methods for the diagnosis and therapy of some liquid tumor types, such as leukemia, which develops slowly, have also been developed. Leukemia has the ability to invade the blood and lymphatic systems, allowing it to spread throughout the body and remain localized in certain regions, such as the bone marrow. Although the potential advantages of nanotechnology are presented in the current article, expected outcomes in clinical uses have not yet been satisfactory. Furthermore, understanding the process by which nanomaterials trigger immunological responses is necessary to regulate the immunomodulatory effect of these materials in applications. Therefore, more sophisticated, and up-to-date NPs-based tactics are required to eradicate various cancer types with more efficacy, selectivity, and less toxicity supported with clinical trials.

## Data Availability

No data were used for the research described in the article.
